# Effects of different NPK ratio combinations on growth, physiology, and soil physicochemical properties of daylily seedlings

**DOI:** 10.3389/fpls.2026.1842457

**Published:** 2026-05-20

**Authors:** Tiantian Gao, Zishan Guan, Ting Lv, Yufa Long, Shaowen Zheng

**Affiliations:** College of Horticulture, Shanxi Agricultural University, Taigu, China

**Keywords:** agronomic traits, Datong Huanghua, NPK ratio, physiological indicators, soil nutrients and enzymes

## Abstract

To address the issues of imbalanced fertilizer application ratios and low nutrient use efficiency during the seedling stage of daylily, this study used ‘Datong Huanghua’ as the plant material. A pot experiment was conducted based on local conventional fertilization practices, with seven treatments of different nitrogen-phosphorus-potassium (NPK) ratios. The effects of NPK combinations were systematically investigated from three dimensions: plant growth, physiological characteristics, and soil physicochemical properties. Principal component analysis combined with the membership function method was used to establish a multi-index comprehensive evaluation system, in order to identify the most suitable fertilization scheme under the conditions of this experiment from among the seven tested treatments. The results showed that different NPK ratios had significant effects on the seedling growth, physiological characteristics, and soil physicochemical properties of daylily. Treatment B3 (with an NPK ratio of 2.13:0.34:1.21) exhibited the best overall performance. It significantly improved growth indicators such as plant height and leaf length, optimized photosynthetic parameters and photosynthetic pigment accumulation, promoted the synthesis of soluble sugars and soluble proteins, and coordinated the root-to-shoot ratio. In terms of soil environment, treatment B3 significantly increased the content of available potassium and significantly enhanced the activities of protease and urease; however, this treatment had no significant effect on available phosphorus content or invertase activity. Ratios with high N but low P, or with high N but low K, can disrupt plant metabolic balance and inhibit growth. This study identified the optimal nutrient ratio for daylily at the seedling stage that balances high yield and soil health, providing a theoretical basis and technical reference for precision fertilization of daylily.

## 1 Introduction

Daylily (Hemerocallis citrina Baroni), also known as golden needle vegetable or nepenthes, is a perennial herbaceous plant of the genus Hemerocallis in the family Asphodelaceae. It is regarded as a functional vegetable crop due to its ornamental, nutritional, and medicinal values (Hou et al., 2017). China is the place of origin as well as the major production area of this species, with abundant germplasm resources, accounting for more than 90% of the global cultivation area (Li and Dong, 2009). It is mainly produced in Datong of Shanxi, Dali of Shaanxi, Qingyang of Gansu, Qidong of Hunan, and other regions, among which Datong is the largest production area in China with a long cultivation history (Li et al., 2021). Daylily is consumed for its flower buds, along with shiitake mushrooms, wood ear mushrooms, and winter bamboo shoots, it is counted among the ‘Four Great Vegetarian Delicacies’ (Qin et al., 2022), it has high nutritional value, rich in carbohydrates, protein, calcium, phosphorus, iron, carotene, and various vitamins, it offers a range of health benefits, including antioxidant properties, sleep-promoting effects, and antidepressant properties (Zhao et al., 2024).

However, key technical bottlenecks still exist in current daylily production. The seedling stage is a critical period for plant morphogenesis and the refinement of physiological functions, and the growth status during this stage directly determines subsequent yield and quality. Nevertheless, problems such as blind fertilization ratios and imbalances in NPK application rates are common in production practice. Most growers rely on empirical fertilization, which can easily lead to excessive seedling growth, reduced stress resistance, and poor root development in daylily. Simultaneously, it causes soil nutrient imbalances and disruption of enzyme activities. This not only restricts the growth quality at the seedling stage but also poses potential risks for subsequent yield reduction and quality deterioration. Although the regulatory effects of different NPK fertilization combinations on the seedling growth of various crops, such as Phoebe bournei, Chinese fir, and wheat, have been widely demonstrated (Wang et al., 2022; Lai et al., 2021; Long et al., 2018), research on optimizing NPK ratios for the seedling stage of daylily in the main production area of Datong remains scarce. Systematic studies on how different NPK ratios synergistically regulate the agronomic traits and photosynthetic physiological characteristics of daylily seedlings, as well as their effects on rhizosphere soil nutrient availability and enzyme activities, are still weak. Consequently, there is a lack of scientific basis for precise fertilization at the seedling stage, which hinders the high-quality and efficient development of the daylily industry. Therefore, conducting experiments on different NPK ratios during the seedling stage of daylily and screening the optimal fertilization scheme have important theoretical and practical significance for solving practical production problems and enhancing the competitiveness of the industry.

Plant growth and development is a process of synergistic interaction between external morphogenesis and internal physiological metabolism. In daylily production, morphological indicators such as plant height, stem diameter, and crown width reflect vegetative growth status. Leaf length, leaf width, and leaf number determine the photosynthetic area, which serves as the basis for matter production. Photosynthetic parameters and chlorophyll content reflect the plant’s light energy conversion efficiency and photosynthetic potential, while root activity affects the aboveground material supply. Osmotic regulatory substances such as soluble protein and soluble sugar play key roles in maintaining cell turgor pressure and responding to environmental changes. Nitrogen (N), phosphorus (P), and potassium (K), as the three essential macronutrients for plant growth and development, play a central role in crop cultivation and management. A scientific NPK ratio not only meets the nutrient demands of plants at different growth stages but also serves as one of the most direct and effective cultivation practices for regulating plant growth and optimizing morphogenesis (Deng et al., 2019). Meanwhile, soil nutrients and enzyme activities, as core indicators reflecting soil fertility, respond directly to fertilization practices and thereby indirectly affect plant growth (Liu, 2004).

Based on the above analysis, the following hypotheses were proposed in this study: (1) An appropriate NPK ratio can significantly promote the optimization of agronomic traits such as plant height, stem diameter, and crown width of daylily seedlings, and enhance physiological indicators including photosynthetic rate, chlorophyll content, and root activity; (2) Rational combined application of NPK can increase the contents of available N, P, and K in the soil, enhance the activities of soil enzymes such as protease, sucrase (invertase), and urease, and regulate the rhizosphere growing environment; (3) There exists an optimal NPK ratio that balances the seedling growth, physiological characteristics, and soil fertility of daylily, under which the contents of soluble sugars and soluble proteins in the seedlings are significantly higher than those under other treatments.

**Figure 1:**
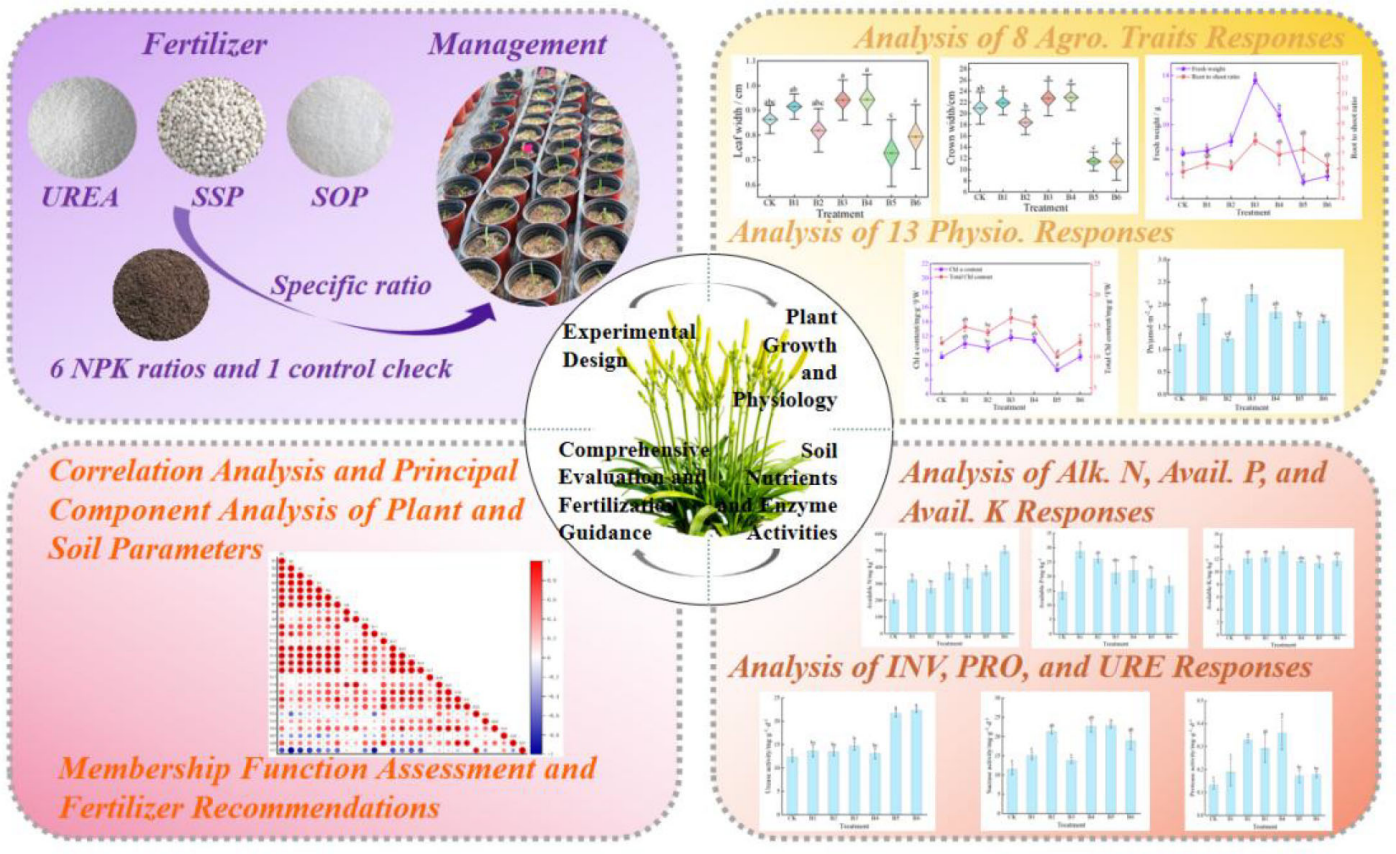
Schematic diagram of the technical approach in this work.

This study adopted a pot experiment to systematically measure eight agronomic traits, thirteen physiological indicators, three soil available nutrient contents, and three soil enzyme activities, and performed correlation analysis on all indicators (**Figure 1**). Systematically evaluate the effects of different NPK ratios on the seedling growth, physiological characteristics, soil nutrient availability, and enzyme activities of daylily, aiming to test the above hypotheses and identify the most suitable NPK ratio for daylily at the seedling stage under the conditions of this experiment, thereby providing a theoretical basis and practical reference for precise fertilization of daylily during the seedling stage.

## 2 Materials and methods

### 2.1 Experimental materials and design

This study used the daylily cultivar ‘Datong Huanghua’ as the plant material. Seedlings were collected from Yunzhou District, Datong City, Shanxi Province (40.02°N, 113.77°E). The experiment was conducted in pots (outer diameter 20 cm, bottom diameter 14.2 cm, height 17.3 cm), with each pot filled with 841.8 g of substrate. The test substrate was a commercial seedling substrate (organic matter content ≥50%, total nitrogen 15 g/kg, total phosphorus 12 g/kg, total potassium 13 g/kg, neutral pH; Shandong Haoze Agricultural Technology Co., Ltd., China). The fertilizers used included urea (46% N, Zhongyan Anhui Hongsifang Fertilizer Co., Ltd., China), superphosphate (7.0% P, converted from 16% P_2_O_5_ × 0.4366, Hubei Fengle Ecological Fertilizer Co., Ltd., China), and potassium sulfate (42.3% K, converted from 51% K_2_O × 0.8298, Suifenhe City Longsheng Economic and Trade Co., Ltd., China).

The experiment was conducted in 2024 at the Horticultural Experiment Station of Shanxi Agricultural University (37.42°N, 112.58°E). The greenhouse environment was controlled as follows: day temperature 25 ± 3 °C, night temperature 18 ± 2 °C, relative humidity 60%-75%, with natural lighting. The local empirical fertilization rate for daylily production (converted to elemental basis: N 13.5 kg/667 m², P 17.5 kg/667 m², K 12.45 kg/667 m²) was used as the baseline, and the fertilizer application rate per plant was converted accordingly. The experiment included three levels of urea: 2.32, 4.64, and 6.96 g per plant; two levels each of superphosphate (2.4 and 4.8 g per plant) and potassium sulfate (2.87 and 5.74 g per plant). After conversion based on the effective nutrient content of the fertilizers, the actual application rates of N, P, and K for each treatment are shown in **Table 1**. For each treatment, the fertilizers were mixed evenly according to the designed ratio and applied in three splits at 40%, 30%, and 30% of the total amount: 40% as basal fertilizer, 30% as the first topdressing, and 30% as the second topdressing. For basal application, the corresponding amount of fertilizer was thoroughly mixed with the substrate in the pot before planting to ensure uniform distribution throughout the rhizosphere soil layer. For topdressing, at 20 days (first topdressing) and 40 days (second topdressing) after transplanting, the corresponding amount of fertilizer was dissolved in water and evenly irrigated around the base of the plants, avoiding direct contact of the fertilizer solution with stems and leaves. Each experimental plot contained 90 plants, with a plot area of 2.5 m² (5 m × 0.5 m), and the experiment was conducted with three biological replicates. For irrigation management, the soil water content was controlled using the weighing method. The initial total mass (pot + substrate) was recorded at the time of potting, and the pots were weighed every two days, with water added to restore 80% of the initial mass (equivalent to maintaining soil water content at 70%-80% of field capacity). Water was slowly applied along the inner wall of the pot to avoid disturbing the substrate surface. During the first 10 days after transplanting, water was appropriately restricted (maintaining 60%-70% of field capacity) to prevent root rot. Except for the fertilization treatments, all other cultivation and management practices were kept consistent, and no fertilizer was directly mixed into the soil.

**Table 1:** N, P, and K application rates for each treatment.

Number	Treatment	N (g/plant)	P (g/plant)	K (g/plant)
CK	N0P0K0	0	0	0
B1	N1P2K2	1.07	0.34	2.43
B2	N2P1K2	2.13	0.17	2.43
B3	N2P2K1	2.13	0.34	1.21
B4	N1P1K2	1.07	0.17	2.43
B5	N3P2K1	3.20	0.34	1.21
B6	N3P1K2	3.20	0.17	2.43

### 2.2 Determination of growth and physiological parameters of daylily

#### 2.2.1 Growth parameters

On the 60th day of the seedling stage, five healthy and uniformly growing plants were randomly selected from each of the three replicate plots per treatment to measure morphological indicators and biomass. Plant height was measured as the vertical distance from the stem base to the tip of the longest leaf using a ruler. Leaf length and leaf width were measured on functional leaves with a ruler. Crown width was determined as the maximum natural spreading width of the plant using a tape measure. Stem diameter was measured at the base of the leaf cluster using a vernier caliper. Leaf number was counted as the number of fully expanded functional leaves. The whole plant was carefully uprooted, and the roots were gently washed and blotted dry of surface water. The fresh weight of aboveground parts (leaves and leaf sheaths) and underground parts (roots) was separately measured using an electronic balance. Total fresh weight and the root-to-shoot ratio (underground fresh weight/aboveground fresh weight) were calculated.

#### 2.2.2 Physiological parameters

On a clear morning between 10:00 and 11:00 on the 60th day of the seedling stage, photosynthetic parameters were measured using a portable photosynthesis system (LI-6400XT, LI-COR, USA). From each of the three replicate plots per treatment, one healthy and uniformly growing daylily plant was randomly selected. The second to third fully expanded functional leaves from the top of the plant were taken, and the net photosynthetic rate (Pn), stomatal conductance (Gs), intercellular CO_2_ concentration (Ci), and transpiration rate (Tr) were measured at the middle portion of the leaf (approximately one-third of the leaf length from the tip) (Long and Bernacchi, 2003).

The leaves used for the determination of chlorophyll, soluble sugar, and soluble protein contents, as well as the root tips used for the root activity assay, were collected from the same batch of plants as those used for the photosynthetic parameter measurements. Each treatment consisted of three replicate plots, and one healthy, uniformly growing plant was randomly selected from each plot, resulting in three biological replicates per treatment. Chlorophyll content was determined using the 95% ethanol extraction method. Fresh leaves (0.2 g) were weighed, cut into pieces, and placed in a test tube, then extracted in the dark for 24 h until the leaf tissue turned completely white. Subsequently, the absorbance was measured at wavelengths of 665 nm, 649 nm, and 470 nm, and the contents of chlorophyll a (Chl a), chlorophyll b (Chl b), total chlorophyll (Chl s), and carotenoids (Car) were calculated separately (Lichtenthaler and Wellburn, 1983).

Root activity was determined by the TTC method. Root tip sample (0.5 g) was weighed and incubated with TTC solution and phosphate buffer in the dark at 37 °C for 2 hours. The reaction was terminated by adding sulfuric acid, and the product was extracted with ethyl acetate. The absorbance was measured at 485 nm (Clegg, 1956).

Soluble sugar content was determined using the anthrone colorimetric method. A 0.1 g dry sample was weighed, extracted in a boiling water bath, filtered, and diluted to a constant volume. Anthrone reagent was then added for color development, and the absorbance was measured at 620 nm (Bradford, 1976).

Soluble protein content was determined using the Coomassie Brilliant Blue G-250 staining method. A 0.2 g fresh sample was weighed, ground with distilled water to form a homogenate, and centrifuged. The supernatant was collected, Coomassie Brilliant Blue reagent was added, and the absorbance was measured at 595 nm. Bovine serum albumin was used as the standard for quantification (Richter et al., 2007).

### 2.3 Determination of soil physicochemical indicators

To investigate the effects of fertilization treatments on soil properties, potting substrate samples were collected on the 60th day of the seedling stage to determine nutrient content and enzyme activity (Wu et al., 2022). The potting substrate corresponding to the three biological replicate plants used for physiological indicator measurements was collected as samples (three soil samples per treatment). After removing the surface debris, a multipoint mixed sampling method was used to collect substrate from the 2–15 cm rhizosphere soil layer in each pot. The samples were mixed, gravel and visible root residues were removed, and then placed in sterile bags and quickly transported to the laboratory. One portion of the fresh soil sample was passed through a 2 mm sieve and stored at 4°C for enzyme activity determination, while the remaining sample was air-dried, ground, sieved, and used for nutrient analysis.

Soil alkaline-hydrolyzable nitrogen was determined using the alkaline hydrolysis diffusion method. The soil sample was reacted with boric acid and sodium hydroxide in a diffusion dish, incubated at constant temperature for 24 h, and then titrated with standard acid. Available phosphorus was determined by the molybdenum blue colorimetric method. Soil available phosphorus was determined by the sodium bicarbonate extraction method. The soil sample was extracted with sodium bicarbonate, reacted with molybdenum−antimony color reagent, and measured at 700 nm. Available potassium was determined using the ammonium acetate extraction-flame photometry method. The soil sample was extracted by shaking with neutral ammonium acetate, then filtered. The potassium concentration in the filtrate was determined using a flame photometer.

The determination of soil enzyme activities focused on key enzymes involved in the carbon and nitrogen cycles. Invertase activity was determined using the DNS colorimetric method. For the determination of invertase activity using the DNS colorimetric method, soil samples were incubated with sucrose solution and phosphate buffer at 37 °C for 24 h, after which the mixture was filtered. The filtrate was then heated with DNS reagent, and the absorbance was measured at 508 nm. Urease activity was determined using the indophenol blue colorimetric method. Soil samples were incubated with urea and citrate buffer at 37 °C for 24 hours, then filtered. The filtrate was reacted with sodium hypochlorite and sodium phenate, and the absorbance was measured at 578 nm. Protease activity was determined using the ninhydrin colorimetric method. For the determination of protease activity using the ninhydrin colorimetric method, soil samples were incubated with casein solution and Tris buffer at 50 °C for 2 h, then filtered. The filtrate was heated with ninhydrin reagent for color development, and the absorbance was measured at 500 nm.

### 2.4 Data processing and analysis

Statistical analysis was performed using Excel software (2010, Microsoft Corp., Redmond, WA, USA), further data analysis was conducted using SPSS software (version 27.0, IBM Corp., Armonk, NY, USA). A one-way analysis of variance (ANOVA) model was used to test overall differences. When significant differences among groups were detected (P< 0.05), the least significant difference (LSD) method was used for *post-hoc* multiple comparisons. In this study, five biological replicates were set for agronomic traits, and three biological replicates were set for physiological indicators, soil nutrients, and soil enzyme activities. Principal component analysis (PCA) was performed using the seven experimental treatments as samples and 27 indicators as variables. The raw indicator data were first standardized to eliminate the effects of different units, as shown in Equation 1. A correlation matrix among the indicators was constructed based on Pearson linear correlation coefficients, and PCA was conducted accordingly. The variance contribution rate and cumulative contribution rate were used to characterize the efficiency of information extraction. The analysis was used solely for comparing overall differences among treatments. Graphs were created using Origin software (2015, OriginLab Corp., Northampton, MA, USA). Comprehensive evaluation was conducted using the membership function method (Su et al., 2023).

(1) $$µ\left(X_{j}\right)=\left(X_{j}-X_{min}\right)/\left(X_{max}-X_{min}\right),\;j=1,\;2,\;3,\;\ldots ,\;n$$

Where X_j_ represents the score of the j-th principal component in a given principal component; X_min_ and X_max_ represent the minimum and maximum scores of a given principal component obtained from principal component analysis. Formula for weight calculation formula is shown in Equation 2:

(2) $$Wj=Pj/\;\overset{n}{\underset{j=1}{\sum }}Pj,j=1,2,\text{...},n$$

Where W_j_ represents the importance (i.e., weight) of the j-th comprehensive index among all evaluation indexes; P_j_ represents the contribution rate of the j-th comprehensive index for each daylily treatment obtained from principal component analysis, Calculation formula for the comprehensive evaluation D value is shown in Equation 3:

(3) $$D=\sum_{j=1}^{n}\left[u(Xj)\times Wj\right]=1,2,\text{...},n$$

Where D value represents the comprehensive evaluation score of each daylily treatment assessed based on 27 indexes. A higher D value indicates better comprehensive performance.

## 3 Results

### 3.1 Effects of NPK ratios on the vegetative growth of daylily

**Figure 2:**
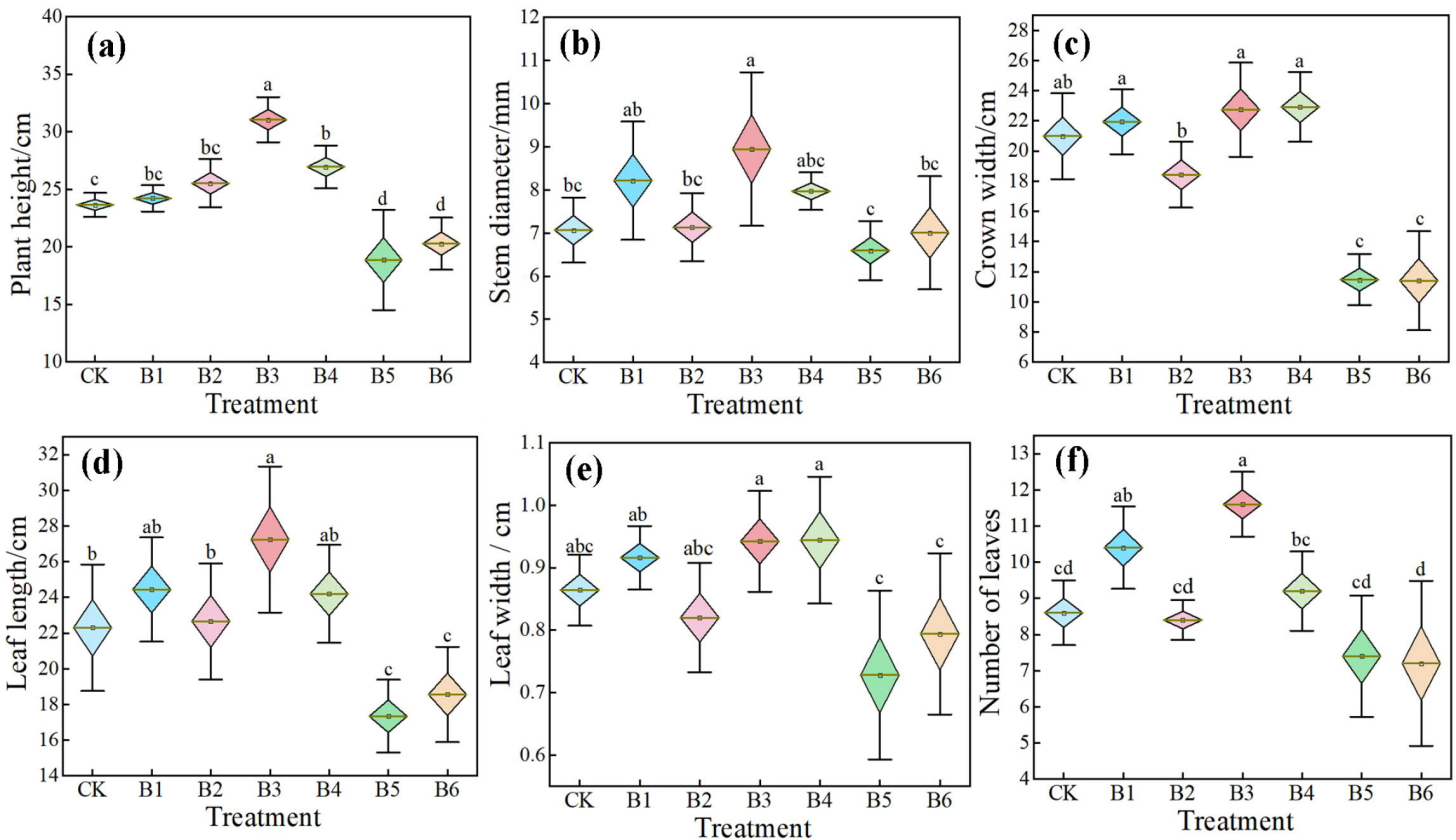
Effects of NPK ratios on the agronomic traits of daylily. **(a)** Plant height; **(b)** Stem diameter; **(c)** Crown width; **(d)** Leaf length; **(e)** Leaf width; **(f)** Number of leaves. Different lowercase letters indicate significant differences at P < 0.05, same as below.

Different NPK ratios significantly regulated the agronomic traits of daylily at the seedling stage, with clear differences among treatments (**Figure 2**). Overall, treatment B3 exhibited the most outstanding performance: plant height, leaf length, and leaf number increased significantly by 31.30%, 23.95%, and 34.88%, respectively, compared with the control (CK); stem diameter, crown width, and leaf width also increased by 26.45%, 10.45%, and 9.03%, respectively. Although the latter three differences were not significant, an overall trend of comprehensively promoting vegetative growth was observed, indicating that this ratio is conducive to optimizing source-sink relationships and enhancing photosynthetic production potential. Treatment B1 also showed a certain positive regulatory effect, with a significant increase in leaf number of 20.93%. The other traits, such as plant height and stem diameter, increased by 2.37% to 16.12%, though not significantly, reflecting the positive role of phosphorus and potassium in leaf differentiation and plant architecture formation. However, under conditions of relatively insufficient nitrogen, the overall growth-promoting effect was constrained. In treatment B4, only plant height significantly increased by 13.96%, while the other traits showed increases ranging from 6.98% to 12.59%, none of which were significant. Treatment B2 had a weak effect on most traits: although plant height and leaf length increased slightly (by 7.95% and 1.61%, respectively), crown width and leaf width decreased by 9.52% and 5.09%, respectively, suggesting that insufficient phosphorus may limit leaf expansion and canopy development.

In contrast, the high-nitrogen treatments B5 and B6 markedly inhibited plant growth, with most indicators being lower than those of the control. The decrease in crown width was the most pronounced, reaching 44.27% and 46.55%, respectively. Plant height, leaf length, and leaf number also significantly decreased by 13.95% to 22.24%, while stem diameter and leaf width showed decreasing trends as well. Thus, imbalance or deficiency in NPK supply, especially nitrogen deficiency or the lack of other elements under high-nitrogen conditions, severely restricts the morphogenesis and biomass accumulation of daylily at the seedling stage.

**Figure 3:**
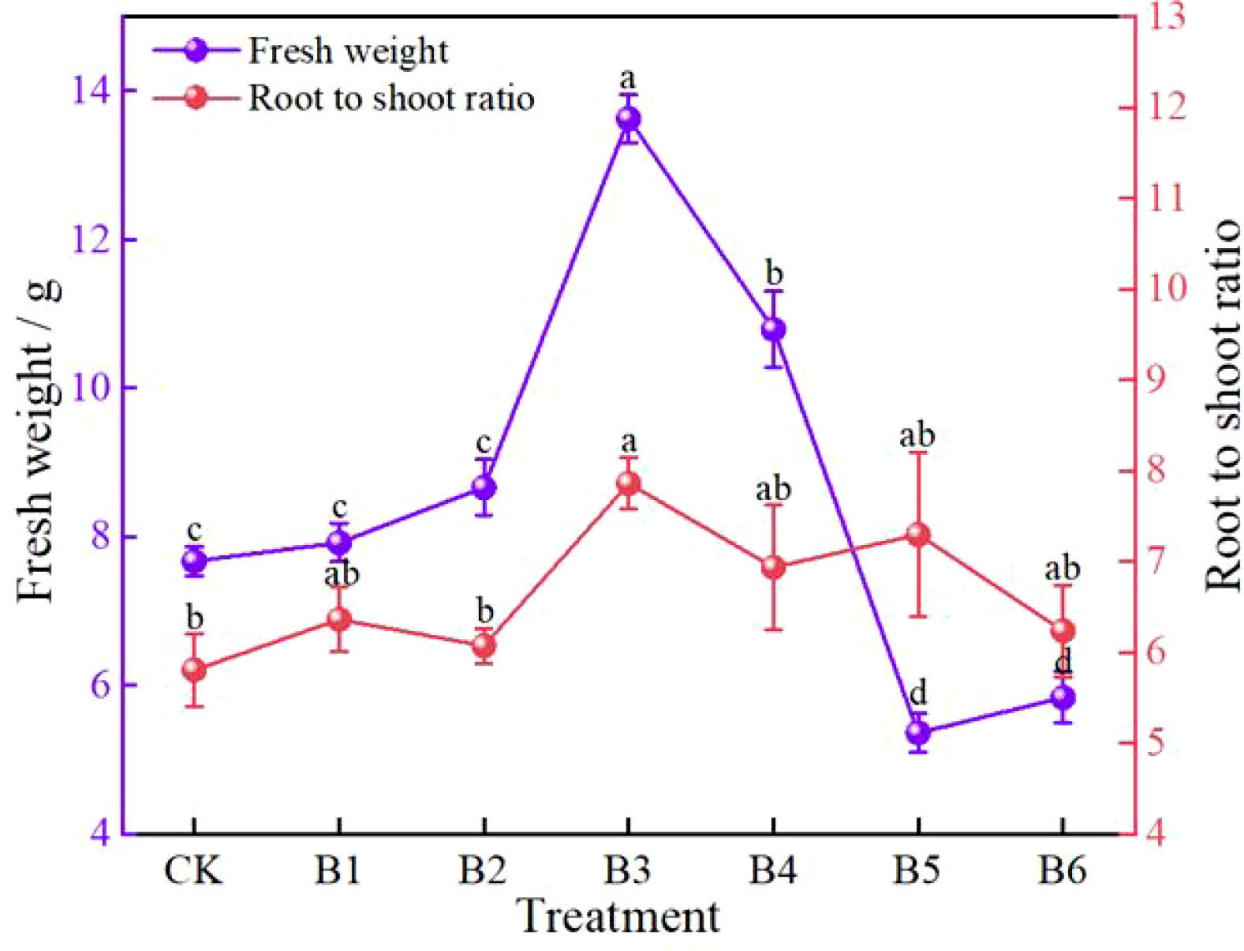
Effects of NPK ratios on fresh weight and root-to-shoot ratio of daylily. Different lowercase letters indicate significant differences at P < 0.05, same as below.

In terms of plant fresh weight, there were significant differences among treatments (**Figure 3**). Treatment B3 had the highest fresh weight, which was significantly increased by 77.71% compared with CK, exhibiting the strongest growth-promoting effect. Treatment B4 ranked second, with a significant increase of 40.68%, also showing a relatively favorable effect. Treatments B1 and B2 had slightly higher fresh weights than CK, but the differences were not significant. In contrast, the fresh weights of treatments B5 and B6 were lower than CK, with significant decreases of 30.12% and 24.02%, respectively, indicating that ratios with high nitrogen but low potassium or high nitrogen but low phosphorus are unfavorable for biomass accumulation.

The change in root-to-shoot ratio showed a different trend from that of aboveground parts (**Figure 3**). The root-to-shoot ratio of all fertilization treatments was higher than that of CK. Among them, treatment B3 significantly increased the ratio by 35.52%, indicating that this ratio not only promoted aboveground growth but also coordinated root development well, favoring overall structural optimization. Although the root-to-shoot ratios of the other treatments (B1, B2, B4, B5, and B6) were also increased, the increases were small and not significant, suggesting that these ratios had a relatively limited regulatory effect on biomass allocation.

### 3.2 Effects of NPK ratios on the physiological characteristics of daylily

#### 3.2.1 Changes in photosynthetic parameters

**Figure 4:**
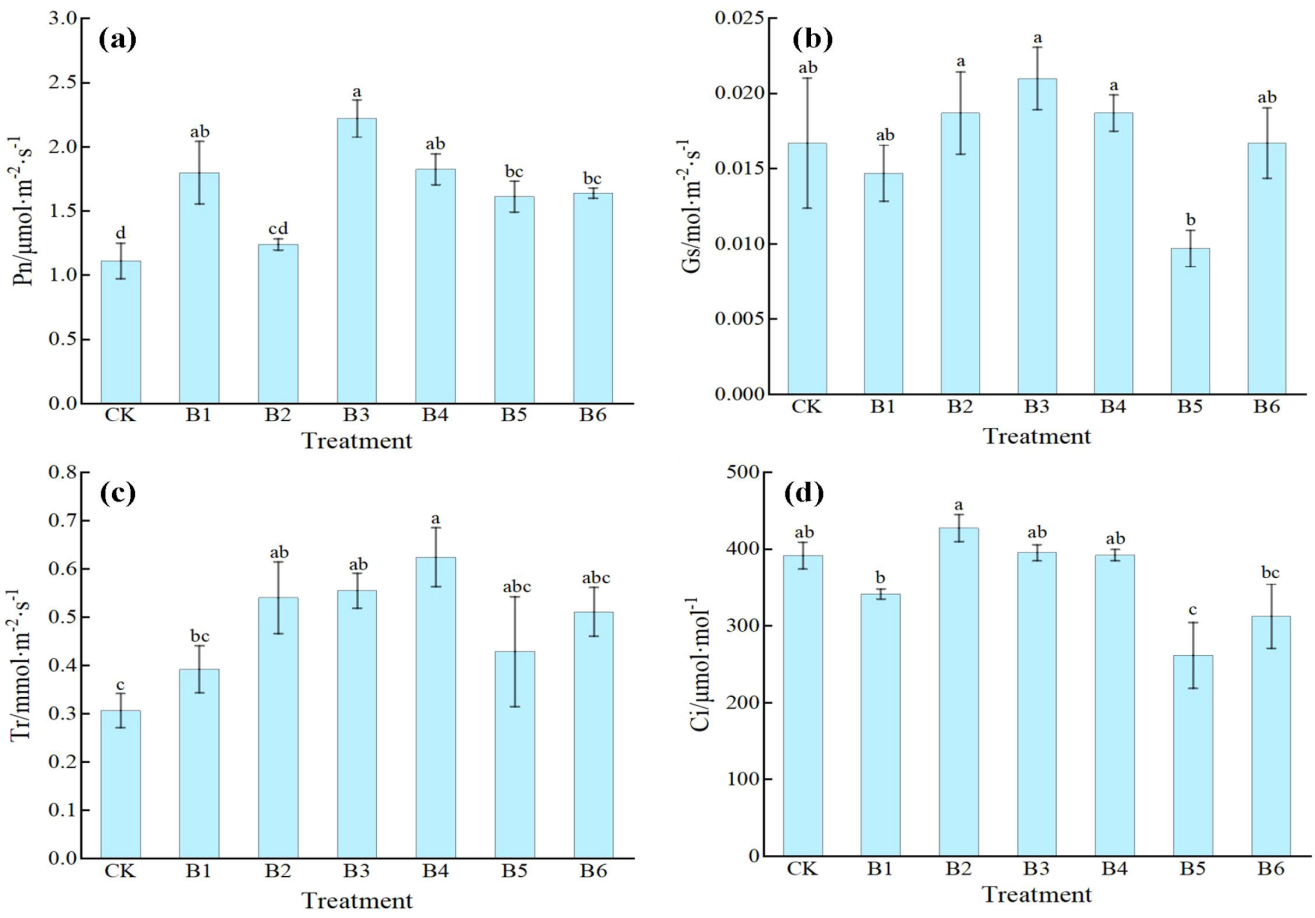
Effects of NPK ratios on the photosynthetic parameters of daylily. **(a)** Pn, Photosynthetic rate; **(b)** Gs, Stomatal conductance; **(c)** Tr, Transpiration rate; **(d)** Ci, Intercellular CO2 concentration. Different lowercase letters indicate significant differences at P < 0.05, same as below.

**Figure 4** shows that different NPK combination treatments significantly affected the photosynthetic parameters of daylily leaves, with each indicator responding differently to the ratios. For net photosynthetic rate (Pn), all treatments except B2 were significantly higher than CK. Among them, treatment B3 exhibited the most prominent effect, increasing Pn by 100% compared with CK. Treatments B1, B4, B5, and B6 also showed significant increases of 62.16%, 64.86%, 45.05%, and 47.75%, respectively. Although treatment B2 was slightly higher than CK, the difference was not significant. The change pattern of stomatal conductance (Gs) was generally consistent with that of Pn: treatment B3 was significantly higher than CK by 25.75%; treatments B2 and B4 also showed significant increases of 11.98%; whereas treatments B1, B5, and B6 showed no significant differences from CK, indicating that an appropriate NPK ratio effectively promotes stomatal opening. For transpiration rate (Tr), treatments B2, B3, and B4 were significantly higher than CK, with increases of 74.19%, 80.65%, and 100%, respectively; treatment B4 had the highest transpiration rate. The remaining treatments showed no significant changes compared with CK. Intercellular CO_2_ concentration (Ci) showed relatively small changes; only treatment B2 was significantly higher than CK by 9.24%, while no significant differences were observed for the other treatments, suggesting that fertilization has a relatively limited regulatory effect on intercellular CO_2_ concentration.

Overall, treatment B3 exhibited clear advantages in enhancing net photosynthetic rate and stomatal conductance, whereas treatment B4 was particularly effective in promoting transpiration. Notably, although treatments B5 and B6 increased net photosynthetic rate, their stomatal conductance and transpiration rate did not increase correspondingly, suggesting that the improvement in photosynthetic performance in these treatments may be limited by non-stomatal factors.

#### 3.2.2 Changes in leaf photosynthetic pigment content

**Figure 5:**
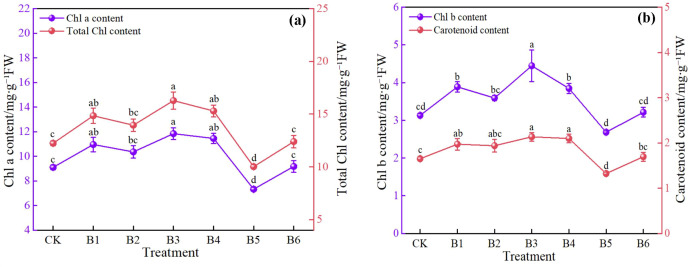
Effects of NPK ratios on leaf photosynthetic pigments of daylily. **(a)** Chl a, Chlorophyll a and Total Chl, Total Chlorophyll; **(b)** Chl b, Chlorophyll b and carotenoids (Car). Different lowercase letters indicate significant differences at P < 0.05, same as below.

As shown in **Figure 5**, different NPK combination treatments significantly affected photosynthetic pigment contents, and the response patterns of each pigment indicator to the different ratios were generally consistent. Regarding chlorophyll a content, treatments B1, B2, B3, and B4 were significantly higher than CK, with increases of 20.44%, 13.85%, 30.11%, and 25.82%, respectively. Among them, treatment B3 exhibited the most pronounced promoting effect. In contrast, treatment B5 showed a significant decrease of 19.00%, while treatment B6 showed no significant difference from CK. The change pattern of chlorophyll b content was similar: treatments B1, B2, B3, and B4 significantly increased by 24.28%, 14.70%, 42.17%, and 22.68%, respectively, with treatment B3 again showing the largest increase; treatment B5 significantly decreased by 14.00%, and treatment B6 showed no significant difference. Carotenoid content followed the same trend: treatments B1, B2, B3, and B4 significantly increased by 19.39%, 17.00%, 29.10%, and 27.27%, respectively, with treatment B3 showing the greatest increase; treatment B5 significantly decreased by 20.00%, and treatment B6 showed no significant difference. The change in total chlorophyll content further confirmed this pattern: treatments B1, B2, B3, and B4 significantly increased by 21.24%, 14.97%, 33.00%, and 25.00%, respectively, with treatment B3 having the highest content; treatment B5 significantly decreased by 18.21%, and treatment B6 showed no significant difference.

In summary, treatment B3 exhibited the best performance in promoting photosynthetic pigment accumulation, while treatments B1, B2, and B4 also showed varying degrees of improvement, indicating that appropriate NPK ratios can effectively promote pigment synthesis. In contrast, treatment B5 significantly inhibited pigment accumulation, and treatment B6 had no significant effect. A reasonable NPK combined application is key to maintaining and enhancing the photosynthetic pigment content in daylily leaves.

#### 3.2.3 Changes in root activity

**Figure 6:**
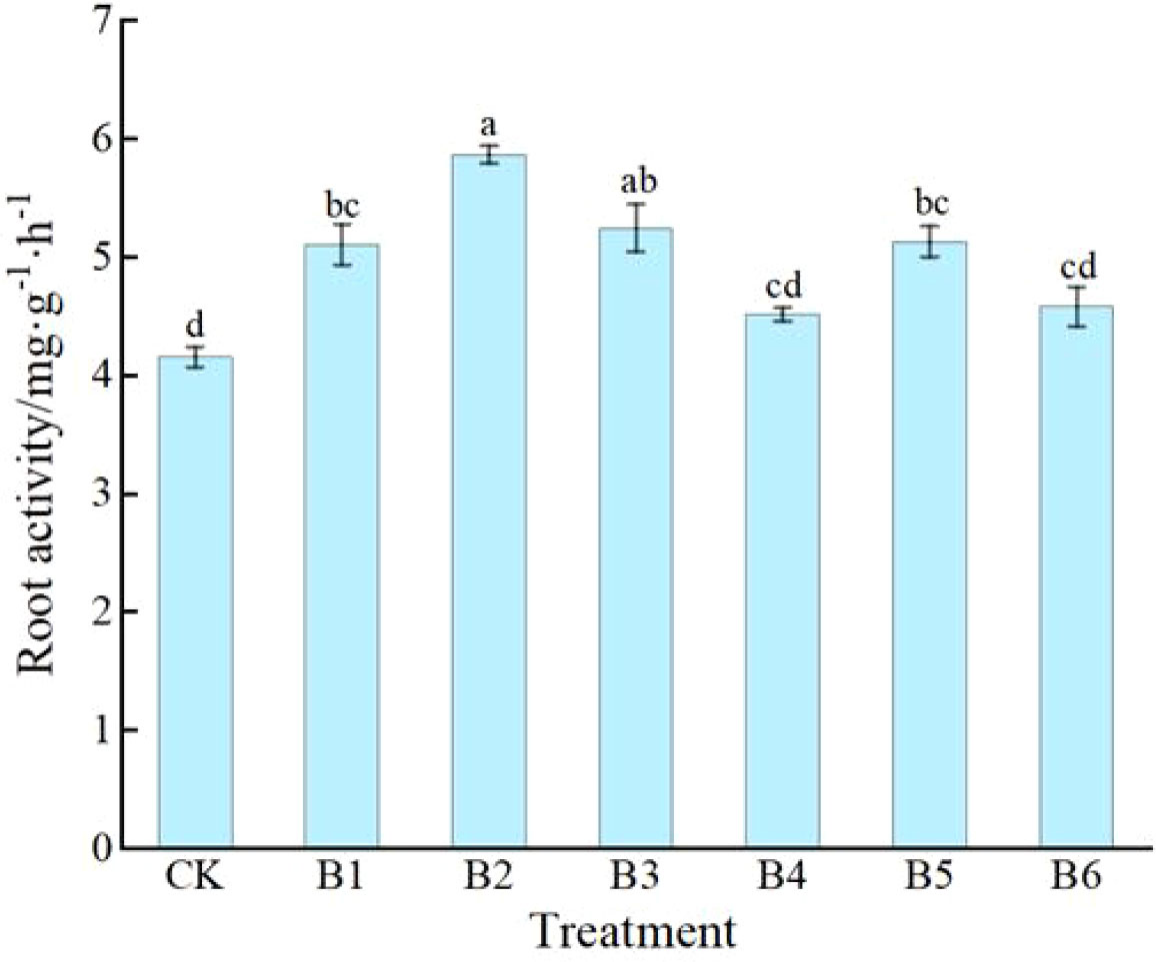
Effects of NPK ratios on root activity of daylily. Different lowercase letters indicate significant differences at P < 0.05, same as below.

**Figure 6** shows that all fertilization treatments promoted root activity in daylily seedlings, although the degrees of increase varied. Compared with CK, treatments B1, B2, B3, and B5 significantly increased root activity, with increases of 22.84%, 41.11%, 26.20%, and 23.32%, respectively. Among them, treatment B2 exhibited the most prominent promoting effect, with an increase exceeding 40%. Treatments B3 and B5 showed intermediate effects, both with increases above 20%, indicating generally strong root metabolic capacity. In contrast, although treatments B4 and B6 were slightly higher than CK, the differences were not significant, suggesting that these two ratios had a relatively limited regulatory effect on root activity. Overall, treatment B2 was the most effective in enhancing root activity, implying that an appropriate NPK ratio, especially with a suitable proportion of potassium supply, may play a positive role in promoting root metabolic activity.

#### 3.2.4 Changes in soluble sugar content

**Figure 7:**
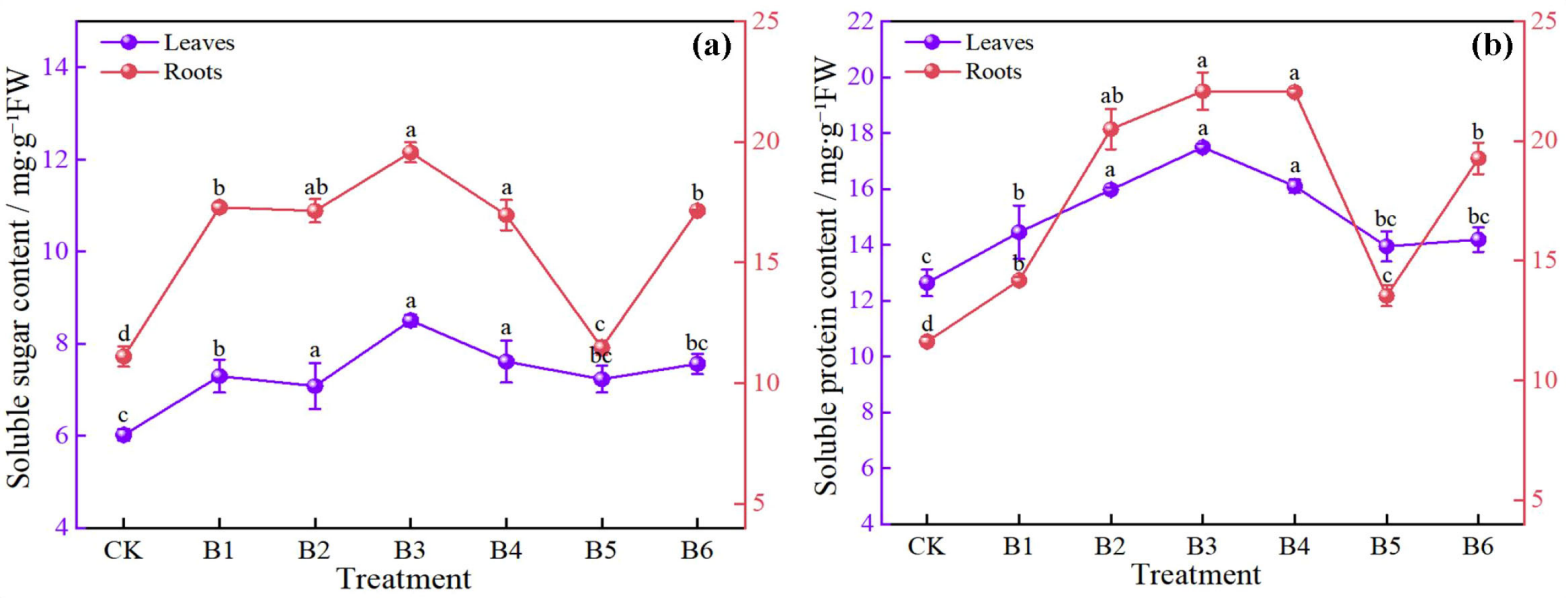
Effects of NPK ratios on soluble sugar and soluble protein contents of daylily. **(a)** leaf and root soluble sugar content; **(b)** leaf and root soluble protein content. Different lowercase letters indicate significant differences at P < 0.05, same as below.

**Figure 7** shows that different NPK combination treatments increased soluble sugar content in both leaves and roots, but the responses differed between organs. In leaves, the soluble sugar content of all fertilization treatments was significantly higher than that of the control (CK), with increases ranging from 17.61% to 41.20%. Among them, treatment B3 had the highest content, representing a significant increase of 41.20% compared with CK, indicating the strongest promoting effect. Treatments B4, B6, B1, B5, and B2 showed intermediate effects, with increases of 26.41%, 25.58%, 21.10%, 20.10%, and 17.61%, respectively. In roots, except for treatment B5, all other treatments had significantly higher soluble sugar content than CK, with increases ranging from 49.43% to 76.31%. Treatment B3 again ranked first in root soluble sugar content, showing a significant increase of 76.31% over CK. Treatments B1, B6, B4, and B2 increased root soluble sugar content by 55.77%, 54.10%, 52.88%, and 49.43%, respectively. Although treatment B5 was slightly higher than CK, the difference was not significant. NPK combined application effectively promoted soluble sugar accumulation, and the magnitude of increase was generally greater in roots than in leaves, indicating that carbohydrate responses were more sensitive in the root system. Among all treatments, B3 exhibited the best promoting effect in both leaves and roots, whereas B5 had a limited regulatory effect on soluble sugar accumulation in roots.

#### 3.2.5 Changes in soluble protein content

As shown in **Figure 7**, different NPK combination treatments increased soluble protein content in both leaves and roots, with organ-specific differences in the responses. In leaves, the soluble protein contents of treatments B1, B2, B3, and B4 were significantly higher than that of the control (CK), with increases of 14.32%, 26.34%, 38.37%, and 27.37%, respectively. Among these, treatment B3 had the highest soluble protein content. Although treatments B5 and B6 showed slightly higher values than CK, the differences were not significant, indicating that these two ratios had limited promoting effects on leaf protein accumulation. In roots, all fertilization treatments resulted in significantly higher soluble protein contents than CK, with increases ranging from 16.44% to 90.10%. Treatment B3 again ranked first, showing a significant increase of 90.10% over CK. Treatments B4, B2, B6, and B1 showed intermediate effects, with increases of 89.85%, 76.42%, 65.92%, and 22.03%, respectively. Treatment B5 had a relatively smaller increase of 16.44%, but it still reached a significant level. NPK combined application significantly promoted soluble protein accumulation, and the magnitude of increase was generally higher in roots than in leaves, indicating that roots were more sensitive to nitrogen nutrition. Among all treatments, B3 exhibited the best promoting effect in both leaves and roots, whereas B5 and B6 had relatively weak regulatory effects on leaf protein accumulation.

### 3.3 Effects of NPK ratios on soil physicochemical properties

#### 3.3.1 Changes in soil nutrient content

**Figure 8:**
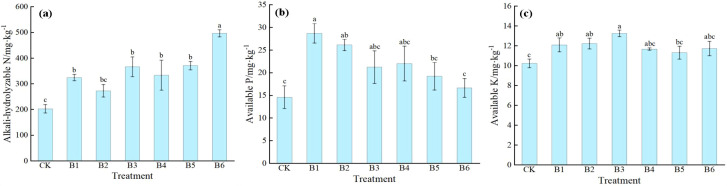
Effects of NPK ratios on soil nutrient of daylily. **(a)** Alkali-hydrolyzable nitrogen content; **(b)** Available phosphorus content; **(c)** Available potassium content. Different lowercase letters indicate significant differences at P < 0.05, same as below.

As shown in **Figure 8**, different combined application treatments of NPK had significant effects on soil nutrient content, and the responses of individual nutrient indicators varied among fertilization ratios. For alkaline-hydrolyzable nitrogen content, all fertilization treatments except B2 showed a significant increase compared with CK. Among them, the B6 treatment had the largest increase, with a significant increase of 144.82% compared with CK, followed by B5, B3, B4, and B1 treatments, with increases of 82.75%, 80.46%, 64.37%, and 59.77%, respectively. The B2 treatment showed no significant difference from CK. For available phosphorus content, only the B1 and B2 treatments showed a significant increase compared with CK, with increases of 96.51% and 79.04%, respectively. No significant differences were observed between the remaining treatments and CK. For available potassium content, B1, B2, and B3 treatments showed a significant increase compared with CK, with increases of 18.28%, 19.55%, and 29.52%, respectively, among which the B3 treatment exhibited the most pronounced increase. B4, B5, and B6 treatments showed no significant difference from CK. Different fertilization treatments had varying regulatory effects on soil nutrients. Treatment B6 resulted in the greatest increase in alkaline-hydrolyzable nitrogen; treatment B1 resulted in the greatest increase in available phosphorus; and treatment B3 resulted in the greatest increase in available potassium.

#### 3.3.2 Changes in soil enzyme activity

**Figure 9:**
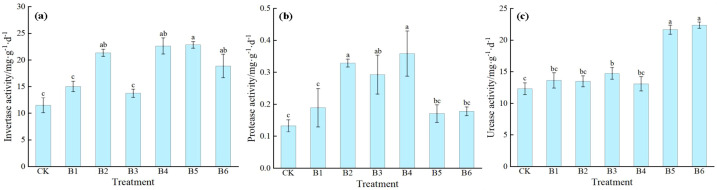
Effects of NPK ratios on soil enzyme activities of daylily. **(a)** Invertase activity; **(b)** Protease activity; **(c)** Urease activity. Different lowercase letters indicate significant differences at P < 0.05, same as below.

**Figure 9** shows that different combined applications of nitrogen, phosphorus, and potassium have significant effects on soil enzyme activities, and the response patterns of individual enzyme activities to the various ratios vary. For invertase activity, treatments B2, B4, B5, and B6 all showed significant increases compared to CK, with increases of 85.24%, 96.53%, 98.26%, and 63.89%, respectively. Among them, treatment B5 showed the most significant promoting effect, while treatments B1 and B3 were not significantly different from CK. For protease activity, treatments B2, B3, and B4 were all significantly higher than CK, with increases of 153.85%, 123.08%, and 176.92%, respectively. Among them, treatment B4 exhibited the highest activity; treatments B1, B5, and B6 were not significantly different from CK. For urease activity, treatments B3, B5, and B6 showed significant increases compared to CK, with increases of 19.55%, 75.75%, and 81.51%, respectively. Among them, treatment B6 exhibited the highest activity; treatments B1, B2, and B4 were not significantly different from CK. The combined application of NPK significantly promoted soil enzyme activities, but the regulatory focus of different treatments varied. Treatment B5 resulted in the greatest increase in sucrase (invertase) activity; treatment B4 exhibited the highest protease activity; and treatment B6 showed the highest urease activity.

### 3.4 Correlation analysis between daylily indicators and soil physicochemical properties

**Figure 10:**
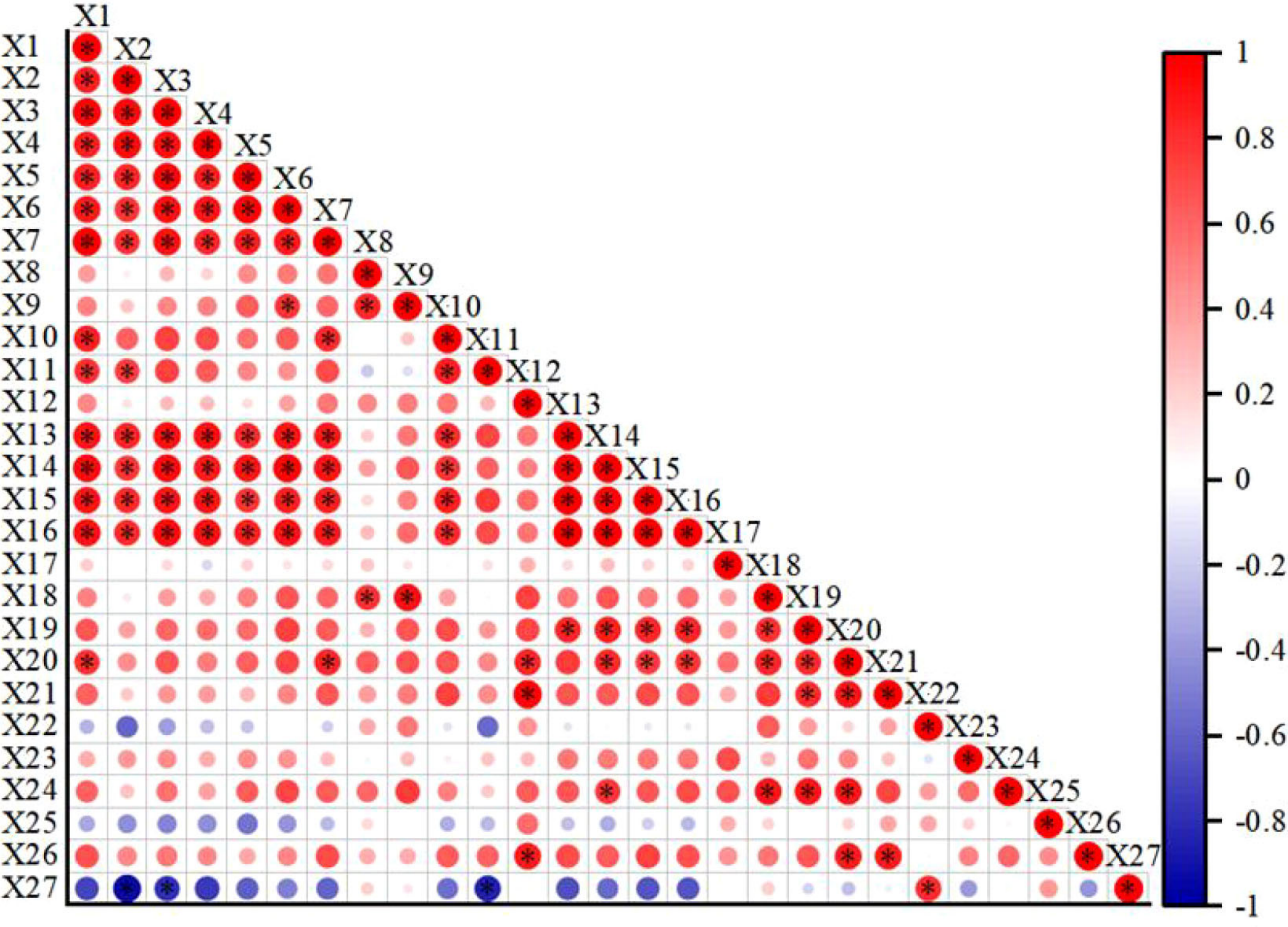
Correlation analysis between daylily indicators and soil physicochemical properties. X1 denotes plant height; X2 denotes crown width; X3 denotes leaf length; X4 denotes leaf width; X5 denotes leaf number; X6 denotes stem diameter; X7 denotes fresh weight; X8 denotes root-to-shoot ratio; X9 denotes Pn; X10 denotes Gs; X11 denotes Ci; X12 denotes Tr; X13 denotes Chl a; X14 denotes Chl b; X15 denotes Car; X16 denotes total chl; X17 denotes root activity; X18 denotes leaf soluble sugar; X19 denotes root soluble sugar; X20 denotes leaf soluble protein; X21 denotes root soluble protein; X22 denotes alkali-hydrolyzable N; X23 denotes Available P; X24 denotes Available K; X25 denotes invertase; X26 denotes protease; X27 denotes urease. *indicates significant correlation at the P< 0.05 level. Red indicates positive correlation, with darker color representing stronger correlation.

The correlation analysis results (**Figure 10**) show that all growth indicators of daylily (plant height, crown width, leaf length, leaf width, leaf number, stem diameter, and plant fresh weight) are significantly positively correlated with each other, indicating that the morphological indicators synergistically promote aboveground biomass accumulation. In terms of physiological indicators, net photosynthetic rate, stomatal conductance, transpiration rate, photosynthetic pigments (chl a, chl b, car, total chl), root activity, as well as soluble sugars and soluble proteins (in leaves and roots), generally exhibited positive correlations with one another. Among them, chl a, chl b, car, and total chl reached a significant positive correlation with soluble sugar content in leaves, indicating that photosynthetic pigments are closely related to the accumulation of photosynthetic products.

In terms of soil physicochemical properties, alkaline-hydrolyzable nitrogen content was positively correlated with available potassium content and invertase activity, and showed a significant positive correlation with urease activity. Available potassium content was positively correlated with most physiological indicators, among which it reached a significant positive correlation with chlorophyll b, leaf soluble sugar, root soluble sugar, and leaf soluble protein content, indicating that potassium plays an important role in promoting the synthesis of photosynthetic pigments and the accumulation of photosynthetic products. Urease activity was positively correlated with root-to-shoot ratio, net photosynthetic rate, leaf soluble sugar content, and invertase activity, and showed a significant positive correlation with alkaline-hydrolyzable nitrogen content. However, urease activity showed a significant negative correlation with most growth indicators (crown width, leaf length) and intercellular CO_2_ concentration, suggesting a possible trade-off between soil nitrogen transformation and aboveground plant growth.

Therefore, appropriate nutrient supply, by coordinating soil enzyme activities and nutrient availability, promotes the improvement of photosynthetic performance and the accumulation of photosynthetic products in daylily, thereby driving plant morphogenesis and biomass increase. Among them, potassium plays a particularly prominent bridging role in coupling the soil-plant system.

### 3.5 Principal component analysis of daylily growth and physiological indicators, and soil physicochemical properties

Principal component analysis (PCA) was performed based on the mean values of all indicators across the seven fertilization treatments. For growth indicators, five biological replicates were used per treatment, while for physiological indicators and soil physicochemical properties, three replicates were used per treatment. The results are presented in **Table 2**. **Table 2** shows the PCA results for daylily growth indicators, physiological indicators, and soil physicochemical properties under different NPK fertilization treatments. Through principal component analysis, a total of five principal components with eigenvalues greater than 1 were extracted, with a cumulative contribution rate of 97.019%, indicating that these five principal components can effectively represent most of the information contained in the original indicators. The first principal component had an eigenvalue of 15.611, with a contribution rate of 57.819%, dominating the analysis. It mainly synthesized information from photosynthetic pigments (chl a, chl b, total chl, car) and plant growth morphology (plant height, fresh weight, leaf length, stem diameter), reflecting the comprehensive level of photosynthetic production capacity and plant biomass accumulation. The second principal component had an eigenvalue of 5.287, with a contribution rate of 19.582%. It was mainly loaded with soil alkaline-hydrolyzable nitrogen, urease activity, and leaf soluble sugar content, reflecting the relationship between soil nitrogen supply status and plant carbon-nitrogen metabolism. The third principal component had an eigenvalue of 2.487, with a contribution rate of 9.210%. It was primarily influenced by soil invertase and protease activities, representing soil biochemical processes and the intensity of nutrient transformation. The fourth principal component had an eigenvalue of 1.706, with a contribution rate of 6.319%. The main determining factors were soil available phosphorus content and root activity, reflecting the synergistic relationship between phosphorus nutrition and root absorption function. The fifth principal component had an eigenvalue of 1.104, with a contribution rate of 4.089%. It was primarily determined by the root to shoot ratio, representing the balance of biomass allocation between aboveground and belowground plant parts.

**Table 2:** Principal component analysis of daylily growth and physiological indicators, and soil physicochemical properties.

Indicator	Principal component				
	1	2	3	4	5
Chl a	0.982	-0.047	-0.129	0.039	-0.122
Total Chl	0.976	-0.115	-0.014	-0.024	-0.150
Chl b	0.966	-0.139	-0.029	-0.047	-0.160
Car	0.958	-0.121	0.116	-0.070	-0.183
Plant height	0.948	-0.210	-0.007	-0.066	0.166
Fresh weight	0.937	-0.096	-0.081	-0.129	0.270
Leaf length	0.917	-0.358	-0.130	0.090	0.071
Stem diameter	0.916	-0.058	-0.386	0.066	-0.029
Leaf soluble protein	0.874	0.408	0.156	0.031	0.166
Leaf number	0.850	-0.236	-0.399	0.238	0.063
Leaf width	0.849	-0.383	-0.198	-0.129	-0.027
Root soluble sugar	0.833	0.361	0.044	-0.012	-0.416
Gs	0.804	-0.181	0.206	-0.431	-0.161
Crow width	0.776	-0.580	-0.027	0.077	0.146
Available K	0.769	0.515	-0.063	0.264	-0.139
Protease	0.752	0.248	0.547	-0.067	0.232
Root soluble protein	0.725	0.494	0.340	-0.333	-0.026
Ci	0.670	-0.515	0.463	-0.161	-0.002
Pn	0.632	0.516	-0.549	0.020	0.080
Alkali-hydrolyzable N	-0.054	0.857	-0.277	-0.248	-0.346
Urease	-0.184	0.760	-0.223	-0.115	-0.116
Leaf soluble sugar	-0.586	0.706	-0.260	0.021	0.015
Invertase	0.650	0.662	0.579	0.052	0.248
Tr	0.607	0.609	0.384	-0.287	0.105
Available P	0.419	0.081	0.264	0.733	-0.218
Root activity	0.506	0.381	0.387	0.694	-0.016
Root to shoot ratio	0.306	0.558	-0.431	0.078	0.561
Eigenvalue	15.611	5.287	2.487	1.706	1.104
Contribution rate	57.819	19.582	9.210	6.319	4.089
Cumulative contribution rate	57.819	77.401	86.611	92.930	97.019

In summary, the differences among the different nitrogen, phosphorus, and potassium ratio fertilization treatments were first reflected in photosynthetic pigment content and plant morphological development; second, in soil nitrogen supply and carbon metabolism; third, in soil enzyme activity, phosphorus availability, and root activity; and finally, in biomass allocation patterns.

### 3.6 Comprehensive evaluation of daylily growth and physiological indicators and soil physicochemical properties

Based on the comprehensive evaluation values (D values) and ranking results of each treatment obtained from principal component analysis (**Table 3**), it was shown that there were significant differences in the overall performance of different nitrogen, phosphorus, and potassium ratio fertilization treatments during the daylily seedling stage. Ranked in descending order of D values, the treatments were as follows: B3 > B4 > B2 > B1 > B6 > B5 > CK. Among them, treatment B3 had the highest comprehensive evaluation value (ranking first in D value), indicating that this NPK ratio exhibited the best overall performance in promoting daylily growth and physiological metabolism while regulating soil physicochemical properties. Treatments B4 and B2 ranked second and third, respectively, also showing relatively prominent comprehensive effects. Treatments B1, B6, and B5 ranked in the middle, with relatively weaker effects. In contrast, the control (CK) had the lowest comprehensive evaluation value. The results further verified the enhancing effect of appropriate NPK combined application on the overall performance of daylily at the seedling stage, with treatment B3 being the optimal fertilization scheme under the conditions of this experiment.

**Table 3:** Comprehensive evaluation and ranking.

Treatment	Principal component score					Comprehensive evaluation value (D value)	Ranking
	Y1	Y2	Y3	Y4	Y5		
CK	5.27	0.81	-0.19	-0.12	-0.22	0.14	7
B1	10.42	1.87	0.90	-0.36	0.11	0.61	4
B2	9.36	2.87	0.77	0.35	-0.61	0.62	3
B3	16.17	2.67	0.06	-0.48	-0.26	0.85	1
B4	12.40	2.47	0.49	0.69	-0.84	0.73	2
B5	3.60	2.19	0.20	0.52	0.20	0.31	6
B6	6.19	2.94	-0.47	0.24	0.18	0.41	5

## 4 Discussion

This study analyzed the effects of different NPK ratios on the growth, physiological characteristics, and soil physicochemical properties of daylily at the seedling stage, preliminarily revealed the response patterns of nutrient regulation, and identified the optimal fertilization scheme under the conditions of this experiment, thereby providing a theoretical basis and technical reference for scientific nutrient management of daylily during the seedling stage.

### 4.1 Regulatory effects of NPK ratios on the vegetative growth of daylily

The growth response of plants is a manifestation of internal physiological metabolism in external morphology. This study confirmed that treatment B3 (NPK ratio of 2.13:0.34:1.21) significantly optimized the morphogenesis of daylily seedlings, with the most pronounced promoting effects on plant height, leaf length, leaf number, and plant fresh weight, which is consistent with the findings of Hu et al (Hu et al., 2022). This balanced NPK ratio can provide a direct nutrient regulation reference for cultivating robust daylily seedlings. Cultivating healthy seedlings is a core prerequisite for ensuring subsequent plant growth and yield potential. From a physiological perspective, balanced nutrient supply positively regulates plant hormone synthesis and accelerates cell division and expansion, providing sufficient material and energy support for plant growth, which is precisely why rational NPK fertilization exerts significant promoting effects on the growth of crops such as daylily and cucumber (Zhao et al., 2015).

It is worth noting that plant requirements for N, P, and K show strong species specificity and vary by growth stage, and no single fertilization ratio is universally optimal. Nutrient input involves a reasonable threshold and inherent trade-offs, where nitrogen, phosphorus, and potassium exhibit both synergistic promotion and antagonistic limitation effects when ratios are imbalanced, and excessive input of any single element cannot sustainably enhance growth. A study by Xue et al. found that nitrogen fertilizer exerted the greatest effect on cabbage biomass, with this effect persisting throughout the entire growth period; the effects of phosphorus and potassium fertilizers were relatively minor (Xue et al., 2017). Wang et al. found that low to moderate nitrogen application rates (0.95-3.79 g/kg) promoted the growth of Allium prattii seedlings, while high nitrogen application rates exerted a significant inhibitory effect (Wang et al., 2024). In this study, treatment B1 with a low nitrogen ratio significantly increased leaf number, but its overall growth-promoting effect was limited. This finding further confirms that insufficient nitrogen supply restricts plant growth, and that increasing nitrogen application is a prerequisite for promoting daylily growth in nutrient-poor soils (Sun et al., 2026). The “3414” fertilizer trial by Gao et al. showed that nitrogen fertilizer had the most significant effect on the growth of daylily, but improper ratios or excessive application can easily lead to negative effects (Gao et al., 2019). In this study, the high-nitrogen treatments B5 (low potassium) and B6 (low phosphorus) inhibited plant growth and underperformed even compared to the control, which is consistent with the findings of Ren et al. that excessive nitrogen application reduces plant biomass. The underlying reason is that excess nitrogen disrupts the allocation balance of carbon assimilation products, impairs root function, and, due to the lack of synergistic effects from phosphorus or potassium, exacerbates metabolic disorders (Ren et al., 2024). Although the high-nitrogen treatments B5 and B6 substantially increased soil available nitrogen levels, plant morphogenesis and biomass accumulation were significantly inhibited, directly reflecting the asynchrony among fertilizer input, soil nutrient surplus, and plant growth and development. In the low-phosphorus treatment B2, crown width and leaf width decreased. In treatments B1 and B2, although soil available phosphorus content increased significantly, aboveground growth and biomass accumulation showed only limited improvement. These observations collectively indicate that an increase in a single soil nutrient does not necessarily translate into proportional benefits for crop growth. Imbalances in the N/K ratio can constrain the synergistic coordination of overall growth, and even when soil basal nutrient reserves are adequate, elemental antagonism may restrict plant morphogenesis. Le et al. found that an appropriate potassium application rate significantly alleviated the inhibition of plant height, stem diameter, chlorophyll content, and photosynthetic efficiency under drought stress, thereby improving plant regrowth capacity (Le et al., 2025). In this study, the significantly increased root-to-shoot ratio in treatment B3 further confirms the key role of potassium in coordinating the balanced development of aboveground and underground plant parts. Maintaining the coordination between aboveground and belowground growth is an important goal of balanced nutrient regulation for ensuring robust plant growth.

### 4.2 Regulatory mechanisms of NPK ratios on the physiological characteristics of daylily

The promoting effect of combined NPK application on daylily growth is essentially achieved through regulating plant physiological metabolism. In this study, treatment B3 exhibited the best overall growth performance, which was closely related to the significant improvement in its photosynthetic physiological indicators. An appropriate NPK ratio significantly increased the net photosynthetic rate and stomatal conductance of daylily, and rational nutrient supply maintained stable stomatal regulation ability of leaves, thereby optimizing leaf gas exchange efficiency. Meanwhile, treatment B3 significantly increased the contents of chlorophyll a, chlorophyll b, and carotenoids in leaves, enhancing the plant’s light capture and utilization capacity, laying the material foundation for efficient photosynthesis, and consequently promoting aboveground growth.

In treatment B4, the transpiration rate was significantly increased. This is attributed to the role of potassium in regulating leaf stomatal movement and water balance, thereby affecting leaf transpiration physiology. In treatments B5 and B6, the net photosynthetic rate increased to some extent, but stomatal conductance and transpiration rate did not change synchronously, and the contents of photosynthetic pigments decreased to varying degrees. A comprehensive analysis based on the measured indicators in this experiment suggests that an excessively high NPK ratio can easily lead to nutrient imbalance, which in turn inhibits photosynthetic pigment synthesis and impairs the coordination of leaf photosynthetic physiology. It is evident that an increase in soil nutrient content does not necessarily benefit plant physiological metabolism; an imbalanced nutrient supply structure can disrupt the coordination of photosynthetic physiology, and excessive nutrient input can have hidden negative effects.

In terms of metabolite accumulation, the soluble sugar and soluble protein contents in both leaves and roots of treatment B3 were significantly higher than those in other treatments, with a more pronounced increase observed in the roots. This indicates that a balanced application of nitrogen, phosphorus, and potassium can promote the synthesis and accumulation of carbohydrates and nitrogenous metabolites. The roots are more sensitive to changes in nutrient supply, and sufficient accumulation of these substances can enhance the physiological stability of the plants. Treatment B2 exhibited the highest root activity but showed only moderate aboveground growth, reflecting a trade-off in the allocation of growth between belowground and aboveground parts. The nutrient composition of this treatment was more favorable for activating root metabolism, yet the overall ratio could not meet the requirements for coordinated canopy growth.

### 4.3 Amelioration effects of NPK ratios on soil physicochemical properties

Soil serves as the primary source of plant nutrients, and its physicochemical properties and biological activity directly influence plant growth and development (Luo et al., 2021). The combined NPK application not only directly regulates physiological metabolism within the plant but also significantly increases the nutrient content of the rhizosphere soil, which is another important pathway through which fertilization promotes crop growth. Numerous studies have shown that combined NPK application can significantly increase soil available nutrient content, thereby providing adequate nutrients for crop growth (Lin et al., 2023). The study by Zhou et al (Zhou et al., 2021) also confirmed that combined NPK application can effectively increase soil alkaline-hydrolyzable nitrogen, available phosphorus, and available potassium contents, and the results of this study are consistent with these findings. All fertilization treatments increased soil nutrient contents to varying degrees, and the regulatory focus on nutrients differed among treatments: treatment B6 preferentially increased available nitrogen, treatment B1 preferentially increased available phosphorus, and treatment B3 preferentially increased available potassium. These patterns are closely related to the nutrient ratios of the respective treatments. It is worth noting that treatment B3 did not receive the highest potassium fertilizer input among all treatments, yet it exhibited the highest soil available potassium content. This phenomenon is not directly determined by the amount of fertilizer applied; rather, it is mainly attributed to the fact that a balanced NPK ratio can promote the activation and release of inherent slowly available potassium in the substrate by regulating the activities of soil enzymes involved in potassium transformation and optimizing the rhizosphere microenvironment, thereby enhancing the soil’s available potassium supply. However, it should also be objectively recognized that persistently high soil available nutrients can easily lead to nutrient accumulation and elemental imbalances, and not all increases in soil nutrients have positive effects. In this experiment, multiple mismatches between nutrient supply and demand were also observed: under high-nitrogen treatments B5 and B6, soil available nitrogen and soil hydrolase activities greatly increased, and the rhizosphere nutrient supply capacity was significantly enhanced; yet plant biomass, photosynthetic pigment accumulation, and vegetative growth were markedly inhibited instead. In treatment B2, soil available potassium accumulated and root activity significantly increased, but aboveground leaf expansion was constrained. In treatment B1, available phosphorus accumulated substantially, yet the overall growth-promoting effect was limited. Long-term, one-sided high-rate fertilization can disrupt the nutrient balance in the rhizosphere (Wang et al., 2016; Zhu et al., 2017; Yang et al., 2021). Furthermore, there is no absolute linear synergistic relationship between soil nutrient content and soil enzyme activity. For example, treatment B3 had the highest available potassium content, yet sucrase activity showed no significant change; in the high-nitrogen imbalance treatments, urease and sucrase activities were abnormally elevated, representing an adaptive fluctuation of the rhizosphere biochemical environment in response to unbalanced nutrient ratios. These observations further indicate that differences in fertilization regimes can lead to a mismatch between soil nutrient status and soil transformation functions.

Soil enzyme activity is an important indicator reflecting soil fertility level and nutrient transformation capacity, and its activity level directly determines the supply efficiency of soil nutrients (Kızılkaya and Hepşen, 2007; Bai et al., 2023). This study found that the activities of soil invertase, protease, and urease in all fertilization treatments were higher than those in the control, which is consistent with the results of numerous previous fertilization experiments (Chen et al., 2015). The long-term field experiment by Wang et al. demonstrated that the combined application of NPK at appropriate ratios significantly increased soil urease and invertase activities (Wang et al., 2008). Shao et al. also pointed out that NPK application synergistically enhanced soil invertase and urease activities and optimized the soil biochemical environment (Shao et al., 2011). Different treatments exhibited distinct regulatory focuses on soil enzyme activities. Some treatments significantly increased invertase activity, while others significantly enhanced protease or urease activity. Among them, treatment B5 showed the most prominent increase in invertase activity, treatment B4 exhibited the best promoting effect on protease activity, and treatment B6 performed optimally in enhancing urease activity. It is worth noting that although treatment B3 significantly activated protease and urease activities, it did not significantly affect invertase activity. This indicates that different NPK ratios can lead to variations in soil enzyme activities, thereby affecting soil nutrient transformation processes and subsequently influencing daylily growth. The soil nutrient pool and soil enzyme activities jointly regulate the release rhythm of rhizosphere nutrients, indirectly affecting root absorption function and the overall physiological status of the plant, reflecting the close relationship between soil biochemical processes and crop growth physiology. In summary, under different NPK combination treatments, there are clear inconsistencies among fertilizer input levels, soil nutrient accumulation characteristics, soil enzyme activities, and plant biomass accumulation. The core reasons lie in the threshold effects of nutrient supply and demand and nutrient antagonism. Under high-nitrogen with low-potassium or low-phosphorus ratios, excessive nitrogen accumulation in the soil can easily lead to an imbalance in carbon-nitrogen metabolism, and excessive inorganic nitrogen increases the metabolic burden on plant cells. Even if soil nutrient pools and enzyme activities are enhanced, the lack of phosphorus-potassium synergy leads to nutrient antagonism, ultimately inhibiting photosynthetic product synthesis and biomass accumulation. In contrast, the balanced ratio of treatment B3, through elemental synergistic effects, simultaneously activates soil nutrient availability and efficient nutrient uptake and utilization by plants, achieving coordinated integration of soil nutrient supply, root absorption, and plant growth metabolism. This is the key to its optimal overall performance. In light of actual production in daylily-growing regions, it is necessary to balance soil fertility maintenance with plant physiological homeostasis. Optimizing nutrient ratios can achieve the synergistic goals of robust seedling cultivation and rhizosphere soil health.

### 4.4 Integrated effects on the daylily-soil system and optimal ratio selection

Correlation analysis revealed a complex network of correlations between daylily growth and physiological indicators and soil physicochemical properties. Among them, available potassium content was significantly positively correlated with chlorophyll b, soluble sugars in leaves and roots, and soluble protein in leaves. This also explains the linkage pattern observed in treatment B3, characterized by low potassium input, high soil available potassium, and high plant potassium accumulation, highlighting the bridging role of potassium in coupling soil nutrient supply with plant photosynthetic metabolism and matter accumulation. Notably, urease activity was significantly positively correlated with alkaline-hydrolyzable nitrogen but significantly negatively correlated with growth indicators such as crown width and leaf length, suggesting a possible trade-off between soil nitrogen transformation and aboveground plant growth. This is speculated to be related to competition for carbon assimilation products between soil nitrogen availability and rapid plant growth. Principal component analysis further clarified the dominant dimensions underlying the differences among fertilization treatments: the first principal component (contribution rate of 57.819%) integrated photosynthetic pigments and plant morphological information, indicating that photosynthetic capacity was the core factor differentiating the treatments. The second principal component (contribution rate of 19.582%) was associated with soil nitrogen supply and plant carbon metabolism. The third to fifth principal components reflected soil enzyme activity, phosphorus availability and root activity, and biomass allocation patterns, respectively. These five principal components systematically elucidated, from different perspectives, the regulatory mechanisms of combined application of nitrogen, phosphorus, and potassium on the daylily soil system.

The comprehensive evaluation results based on principal component analysis showed that among the seven tested treatments, treatment B3 exhibited the best overall performance and was the most suitable fertilization scheme under the conditions of this experiment. Treatments B4 and B2 can be considered alternative options. In contrast, treatments B5 and B6 exhibited poor effects, even worse than the control, suggesting that fertilization patterns with high nitrogen but insufficient phosphorus and potassium (e.g., high N but low K, or high N but low P) should be avoided in production. This provides a direct practical reference for scientific fertilization of daylily at the seedling stage under the conditions of this experiment.

### 4.5 Strengths and limitations of this work

The core strength of this study lies in its integrated analysis from three dimensions: plant growth, physiological metabolism, and soil environment. It clarified the underlying mechanism by which treatment B3 coordinates soil enzyme activities and nutrient availability, enhances photosynthetic efficiency and metabolite accumulation, and optimizes the plant root to shoot ratio, thereby achieving coordinated aboveground and belowground development. This provides both theoretical support and a practical approach for scientific fertilization of daylily at the seedling stage.

This study also has certain limitations, as it only focused on the seedling stage of daylily, while the response patterns to NPK ratios during the full growth period (especially the bolting, budding, and harvesting stages) remain unclear. The experimental results only reflect the effects under specific experimental conditions, and the interactive effects of factors such as climate and soil texture with fertilization in specific ecological regions were not considered. In addition, the long-term effects of long-term site-specific fertilization on soil quality and daylily quality were not addressed. There are also notable limitations in terms of soil nutrient assessment and crop nutrient uptake: only soil available nutrient contents were measured, without converting them into actual crop uptake for quantitative analysis. Crop nutrient uptake involves not only nitrogen, phosphorus, and potassium but also secondary and micronutrients such as calcium and magnesium. This study focused only on NPK ratios and changes in soil fertility; it did not determine secondary or micronutrient contents, nor did it measure plant nutrient accumulation or the patterns of nutrient distribution and transport among different organs. Consequently, the research dimension is narrow, and the nutrient evaluation system remains incomplete. In addition, there were also limitations in the comprehensive evaluation method. First, the weights of the indicators were determined solely based on variance contribution, without considering agronomic importance or physiological significance, which may lead to some indicators with large variability but limited practical production value being assigned excessively high weights. Second, the evaluation model did not distinguish whether an increase in soil nutrient content resulted from improved fertility or from over-fertilization. Third, plant nutrient contents were not measured, making it impossible to quantify nutrient uptake and fertilizer use efficiency. Fourth, the fertilization effects were analyzed using a single-factor design, which did not allow for the analysis of interactions among N, P, and K. Furthermore, this study only included six NPK ratio treatments plus a control, and did not employ continuous optimization models such as full factorial design or response surface methodology. Therefore, the suitable ratio obtained here represents only a relatively better scheme among the tested treatments, and nutrient optimization under broader ratio gradients remains to be further investigated.

Therefore, it is recommended that future studies combine yield components and quality indicators and conduct multi-year, multi-site experiments. It is also suggested that multi-factor experimental designs (e.g., response surface methodology or orthogonal design) be adopted to elucidate the interactive effects of N, P, and K, and that plant nutrient content measurements be included to calculate uptake efficiency. In terms of evaluation methods, priority should be given to weighting strategies based on agronomic importance or production objectives, rather than relying solely on statistical variance. Building on this foundation, future research can further expand the scope of investigation to systematically explore the regulatory effects of the synergy between soil macronutrients and secondary/micronutrients on nutrient uptake in daylily, deeply analyze the transport and distribution patterns of nutrients among roots, stems, leaves, and flowers, as well as the associated enzymatic metabolic response mechanisms, and thereby improve the systematic research framework of “soil fertility—crop nutrient uptake—organ nutrient transport.” Ultimately, further optimization of daylily-specific fertilizer formulas suitable for different ecological regions should be pursued, along with the improvement of the nutrient management technical system for daylily.

## 5 Conclusions

An appropriate NPK ratio can significantly promote the growth of daylily at the seedling stage. Among them, the NPK ratio of 2.13:0.34:1.21 (Treatment B3) exhibited the best overall performance, with significant increases in growth indicators such as plant height, leaf length, and fresh weight, while also optimizing the root-to-shoot ratio and promoting coordinated aboveground and belowground development. This treatment provided a sufficient material and energy basis for plant growth by improving photosynthetic performance (increasing net photosynthetic rate, stomatal conductance, and photosynthetic pigment contents) and promoting the accumulation of soluble sugars and soluble proteins. In addition, rational combined application of NPK significantly regulated soil physicochemical properties and biochemical characteristics. In terms of the soil environment, treatment B3 significantly increased the contents of alkaline-hydrolyzable nitrogen and available potassium, and significantly enhanced the activities of protease and urease, but had no significant effect on available phosphorus content or sucrase activity, thereby improving the overall soil nutrient supply capacity. In contrast, high-nitrogen with low-phosphorus or high-nitrogen with low-potassium ratios tended to disrupt plant metabolic balance and inhibit growth, and such fertilization patterns should be avoided in production practice. In summary, the NPK ratio of 2.13:0.34:1.21 represents a suitable fertilization scheme for daylily at the seedling stage that balances plant growth and soil health, providing a theoretical basis and technical reference for scientific fertilization and nutrient management of daylily during the seedling stage.

## Data Availability

The raw data supporting the conclusions of this article will be made available by the authors, without undue reservation.
